# Rabies Virus Exploits Cytoskeleton Network to Cause Early Disease Progression and Cellular Dysfunction

**DOI:** 10.3389/fvets.2022.889873

**Published:** 2022-05-13

**Authors:** Xilin Liu, Zeeshan Nawaz, Caixia Guo, Sultan Ali, Muhammad Ahsan Naeem, Tariq Jamil, Waqas Ahmad, Muhammad Usman Siddiq, Sarfraz Ahmed, Muhammad Asif Idrees, Ali Ahmad

**Affiliations:** ^1^Department of Hand Surgery, Presidents' Office of China-Japan Union Hospital of Jilin University, Changchun, China; ^2^Department of Microbiology, Government College University Faisalabad, Faisalabad, Pakistan; ^3^Faculty of Veterinary Science, Institute of Microbiology, University of Agriculture, Faisalabad, Pakistan; ^4^Department of Basic Sciences, University College of Veterinary and Animal Sciences, Narowal, Pakistan; ^5^Department of Clinical Sciences, Section of Epidemiology and Public Health, College of Veterinary and Animal Sciences, Jhang, Pakistan; ^6^Department of Clinical Sciences, University College of Veterinary and Animal Sciences, Narowal, Pakistan; ^7^Institute of Microbiology, University of Veterinary and Animal Sciences, Lahore, Pakistan; ^8^Department of Pathobiology, University College of Veterinary and Animal Sciences, Narowal, Pakistan

**Keywords:** Rabies virus, actin, microtubule, phosphoprotein, cytoskeleton, neuron, dynein, endocytosis

## Abstract

Rabies virus (RABV) is a cunning neurotropic pathogen and causes top priority neglected tropical diseases in the developing world. The genome of RABV consists of nucleoprotein (N), phosphoprotein (P), matrix protein (M), glycoprotein (G), and RNA polymerase L protein (L), respectively. The virus causes neuronal dysfunction instead of neuronal cell death by deregulating the polymerization of the actin and microtubule cytoskeleton and subverts the associated binding and motor proteins for efficient viral progression. These binding proteins mainly maintain neuronal structure, morphology, synaptic integrity, and complex neurophysiological pathways. However, much of the exact mechanism of the viral-cytoskeleton interaction is yet unclear because several binding proteins of the actin-microtubule cytoskeleton are involved in multifaceted pathways to influence the retrograde and anterograde axonal transport of RABV. In this review, all the available scientific results regarding cytoskeleton elements and their possible interactions with RABV have been collected through systematic methodology, and thereby interpreted to explain sneaky features of RABV. The aim is to envisage the pathogenesis of RABV to understand further steps of RABV progression inside the cells. RABV interacts in a number of ways with the cell cytoskeleton to produce degenerative changes in the biochemical and neuropathological trails of neurons and other cell types. Briefly, RABV changes the gene expression of essential cytoskeleton related proteins, depolymerizes actin and microtubules, coordinates the synthesis of inclusion bodies, manipulates microtubules and associated motors proteins, and uses actin for clathrin-mediated entry in different cells. Most importantly, the P is the most intricate protein of RABV that performs complex functions. It artfully operates the dynein motor protein along the tracks of microtubules to assist the replication, transcription, and transport of RABV until its egress from the cell. New remedial insights at subcellular levels are needed to counteract the destabilization of the cytoskeleton under RABV infection to stop its life cycle.

## Structural Dynamics of Cytoskeleton

Actin filaments, microtubules, and intermediate filaments are the 3 main components of the cell cytoskeleton. Actin filaments are composed of globular proteins that form small aggregates of actin monomers, and these actin monomers rapidly grow at the plus end of the actin filaments as compared to the minus end through a process called polymerization or nucleation (1, 2). There are 3 isoforms of globular actin, namely, α, β, and γ (2). The globular monomers are connected through weak forces to form long actin filaments. The actin cytoskeleton plays a vital role in viral endocytosis, cell motility, and membrane dynamics (3). The filamentous actin and the pool of globular actin are regulated and maintained by numerous actin-binding proteins (4, 5), while similar kinds of microtubule-binding proteins function to regulate the growth, polymerization, and depolymerization of microtubules (5). The microtubules are made up of α and β polar heterodimers which are arranged in a head-to-tail fashion, forming linear proto-filaments or tubular filaments, whereas 13 protofilaments combine to form microtubules (4, 5). Just like actin, the dynamic growing-end is denoted as the plus or barbed end (having GTP bound β subunit), facing the cell periphery. On the other hand, the depolymerizing or minus end (having α end) is attached to the microtubule organization center in the perinuclear area (4, 6, 7). Tubulins are abundantly present mainly in two pools, the free tubulin subunits and polymers (8, 9). The plus ends of the microtubules show a continuous dynamic state of growth and shrinkage with the intermittent pauses in dendrites, axons, and even in fully matured neurons (10). The microtubules have similar polarity in the axons, but mixed or heterogeneous polarity has been observed in dendrites (7, 11). The neuronal cytoskeleton is a set of complex proteins connected to strengthen the neurons in terms of guidance and growth in an early phase of neuronal development. The development of neuronal architecture, cell shape, homeostasis, and nerve plasticity are accomplished in the adult stage (1, 2, 4, 12). The cytoskeleton also provides support to transport molecular trafficking in the cytosol with the help of motor proteins. The bidirectional movement of molecular cargoes, cellular organelles, and viral genes within the environment of the cytoplasm is termed axonal transport (11). Microtubules extensively support the transport of the membrane-bounded vesicles either in retrograde (from axon to cell body) or anterograde (from cell body to axon) direction (4, 13, 14).

**Figure 1:**
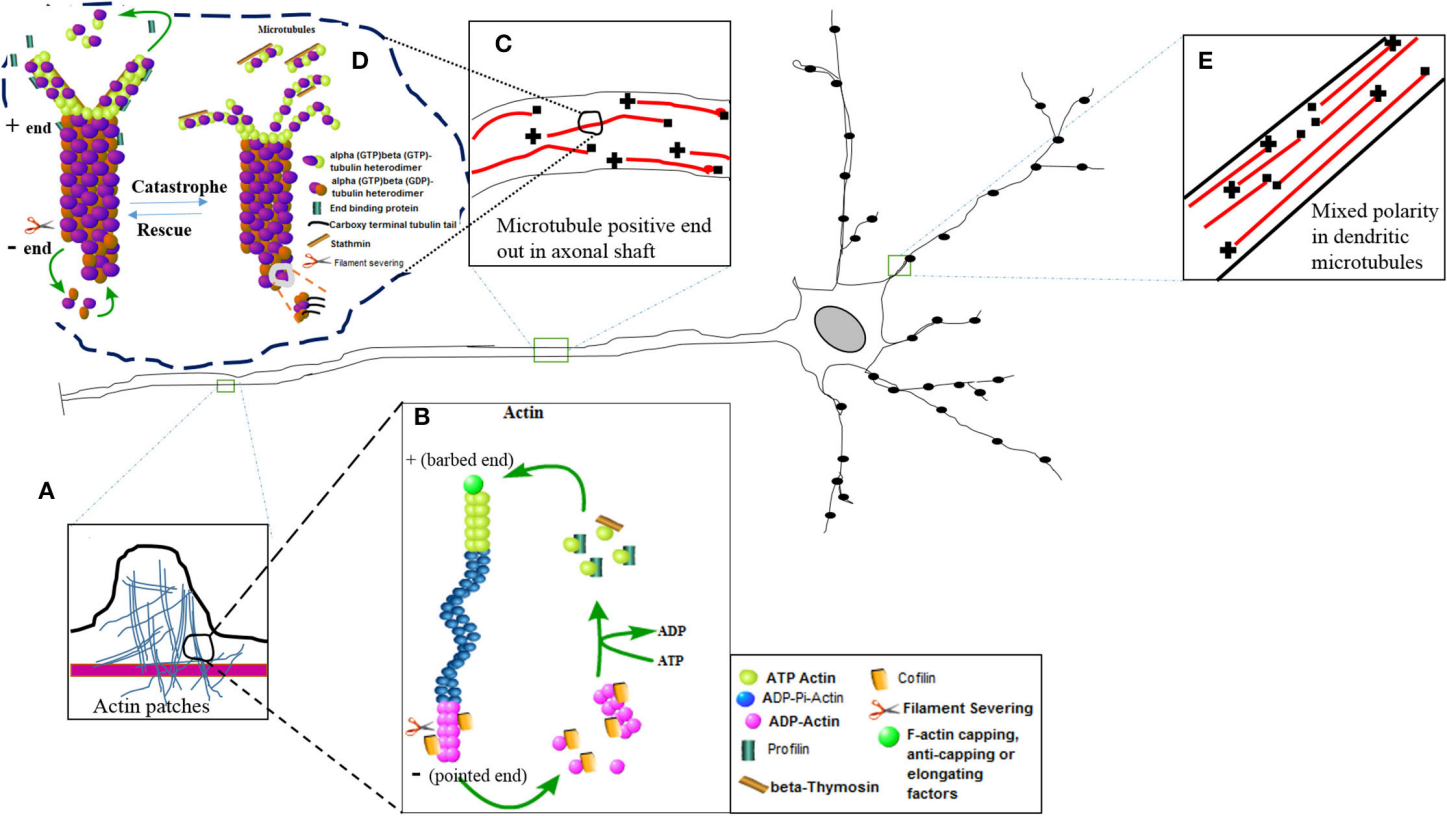
The diagram of nerve cell showing different inset images of the active nature of the actin and microtubule cytoskeleton. **(E)** Mixed polarity of the microtubules exists in the dendrites of the neurons. **(C)** Positive ends are laid down in the axonal shaft for efficient bidirectional axonal transport. **(A)** The actin patches have been magnified in the inset image to show their presence in the axonal shaft. These actin patches act as a source to create further branches arising at different lengths of the axon. Further magnification of actin and microtubule images represent molecular structures showing the active phenomena of actin polymerization **(B)** and microtubule polymerization **(D)** or nucleation with the help of different binding proteins.

The actin and microtubule-binding proteins are the key elements that regulate the shape, structure, and polarity of actin and microtubule filaments, respectively (2, 4). For example, profilin is a noticeable actin-binding protein that is responsible for the binding of actin monomers to complete the cycle of polymerization by catalyzing the exchange of ATP in the actin filaments and thus helps in the growth and elongation of the actin filaments. The shorter nature of the β-thymosin peptides regulates the binding of the globular units (actin monomers) of the actin filaments. It also prevents the attachments of these monomers independently with the minus or plus end of the actin filaments. Cofilin is a vital actin slicing or severing protein that creates the dynamic instability among actin filaments by providing free ends of the actin filaments from which the globular actin monomers can be dissociated or added. The capping proteins cap the plus or barbed ends of the actin filaments and prevent its further elongation by blocking the attachments of globular subunits at the polar ends of actin filaments. Contrary to the roles of capping proteins, the formins proteins normalize the actin dynamics by removing the capping proteins and elongating the actin filaments by exposing the corresponding end binding sites on these filaments to other actin-binding proteins (4, 14, 15). Similarly, the end binding proteins (EB family) of the microtubule are an important set of microtubule-binding proteins that regulate the dynamic nature of the microtubules. The carboxy-terminal tubulin tails (Figure 1) of the tubulin heterodimers coordinate the collective and individual assembly and growth rates of the microtubules. These terminal tails of the heterodimers also present the selective nature of the microtubules to the microtubule-binding proteins. Just like cofilin, stathmin is a microtubule depolymerizing protein (5, 14).

The various functions of these cytoskeleton binding proteins have been shown in Figure 1 in which the dynamic turnover rates of both actin and microtubules have been summarized. This image describes the elongation (polymerization) and shrinkage (depolymerization) of actin and microtubule filaments with the help of these binding partners, and thus regulates the structure, integrity, and biochemical physiology of the nerve cell. Actin treadmilling is a phenomenon in which the actin monomers or individual units of the actin filaments are continuously released from the negative ends and join the positive ends. The profilin adds the ATP actin monomers to the plus ends, while formins uncap the negative ends. The hydrolysis of the ATP actin units occurs, and cofilin removes or slices the units of actin filaments. In this way, the detached actin monomers of the actin filaments present their hydrolyzed ADP in return to the ATP, and once again becomes eligible to perform another cycle of polymerization at the plus end of the actin filaments (15). In a similar fashion, the microtubule heterodimers (alpha and beta heterodimers) attach and detach to nucleate or polymerize the plus end of the microtubules, and hydrolysis of the beta bound GTP tubulin takes place, and similarly a slow rate of nucleation also takes place at the barbed end of the microtubule. The EB family proteins also aid in the process of nucleation. The catastrophic phenomena in the microtubules take place with the help of stathmin, just like the way cofilin does in the actin filaments (5). As a result, the pool of free alpha- and beta-tubulin heterodimers reduces and the length of the microtubule shrinks (Figure 1).

## Cell Biology of the RABV

**Figure 2:**
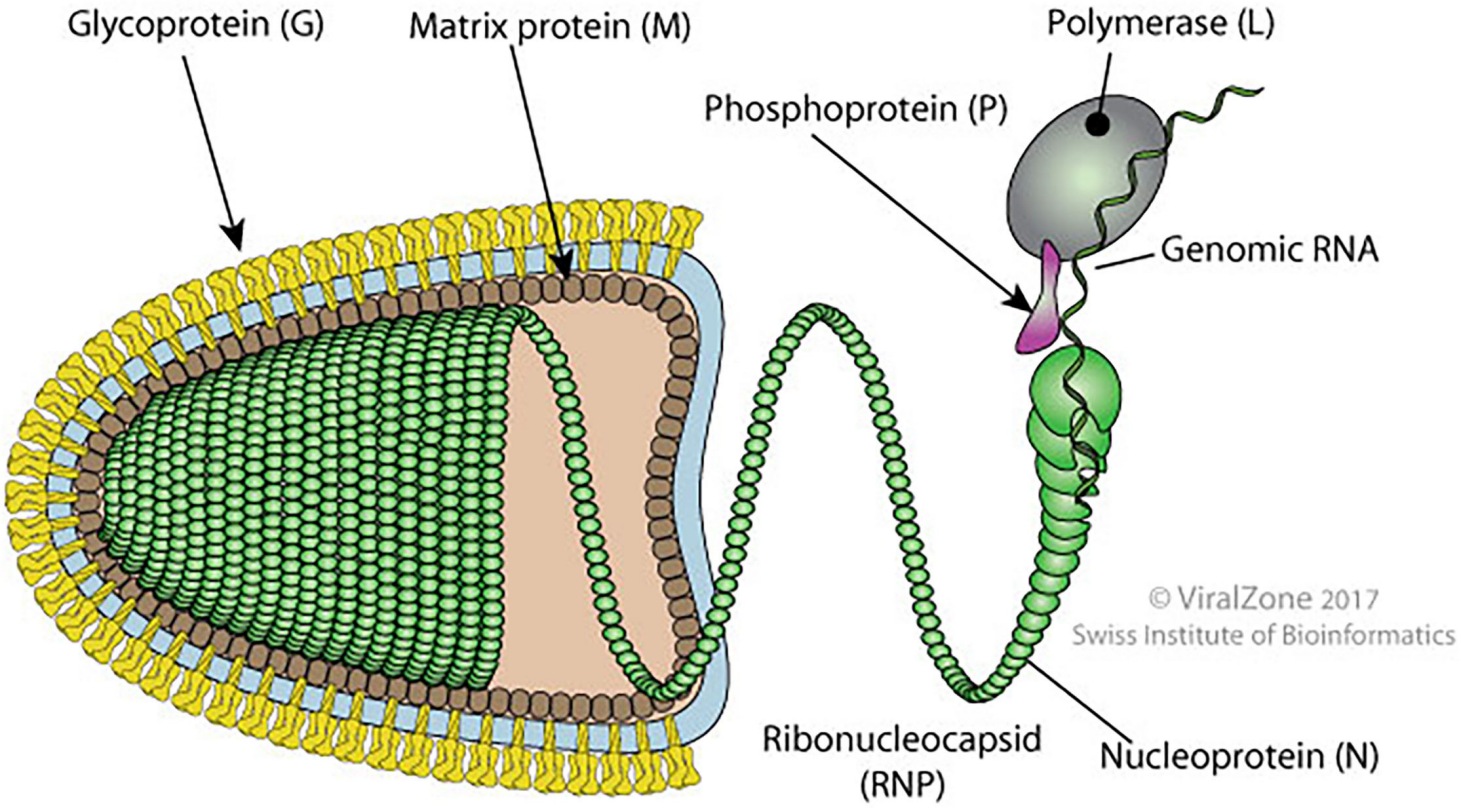
Schematic diagram of bullet shape structure of RABV showing different proteins (original source of the image; Philippe Le Mercier, SIB Swiss Institute of Bioinformatics).

It is a bullet-shaped virus of around 180 nm long and 90 nm diameters. The virus is formed by an internal and an external unit linked together. The internal unit is composed of a nucleocapsid (NC) that includes the genomic RNA tied to the phosphoprotein (P), nucleoprotein (N), and viral polymerase (L). The external unit is formed by protruding spikes of the viral glycoprotein (G) and a bilayer lipid envelope acquired from the host cell membrane. These two units are linked by the matrix protein (M) which interacts with the G protein and condenses the NC (16) (Figure 2).

Rabies virus (RABV) proteins (N, P, M, G, and L) are multifunctional. The N protein ensures the protection of the viral genome from RNAse and is the major component of the NC core. During transcription and replication, the N protein interacts with P and L proteins. The P protein participates in the replication and transcription process as a noncatalytic cofactor for the L protein and disrupts the host interferon-mediated antiviral response (16, 17). During N protein synthesis, the P protein regulates the positioning of the polymerase on the N-RNA template. It also prevents its binding to cellular RNA by acting as a chaperone. The M protein facilitates the budding, apoptosis, and intercellular membrane redistribution (16).

The life cycle of the RABV is dependent upon the clathrin-mediated endocytosis and acidic environment of the cytosol. RABV is coated with a spike-like G protein which is embedded in the lipid layer. The G protein of RABV is an essential indicator to induce pathogenesis inside host cells, and it also helps in the attachment of the RABV to the host cell surface. Moreover, it also assists in the long-distance transport of RABV from the neuromuscular junction to the central nervous system. After the attachment with the host cell receptors, various conformational changes occur in the G protein that uncoats the RABV genome inside cytosol through an endocytic pathway (18). The negative-sense RNA genome is embedded in the N protein and acts as a template for transcription and replication. The ectodomain is the only external section of the G protein which provokes the synthesis of virus-neutralizing antibodies following the immune response mediated by cells. The role of replication and transcription are largely uptaken by the N and P proteins of RABV, while the M protein plays role in budding or viral exit (17–19).

## Materials and Methods

In this comprehensive critical review, a detailed analysis of different RABV proteins and their interactive features with the elements of the cytoskeleton have been discussed through a systematic strategy. All the relevant literature and published scientific research articles have been identified through various phrases and keywords with the help of boolean operators. These keywords and phrases included “Rabies virus and cytoskeleton,” “RABV-Cytoskeleton,” “Rabies virus and microtubule,” “Rabies virus and actin,” “Rabies virus with cytoskeleton binding proteins,” “RABV-actin-microtubule,” “Rabies pathogenesis in cytoskeleton,” “Rabies-actin,” “Rabies-microtubule,” and “RABV depolymerizes cytoskeleton.” In this regard, Google, PubMed, ScienceDirect, and Google Scholar were used as electronic platforms to screen all the possible literature (20). All the materials that described the complete or partial association or interactive feature of RABV (or one of the related proteins of RABV) with the cytoskeleton elements (actin, microtubule, and binding proteins of cytoskeleton) were collected. Meaningful conclusions and interpretations have been made in the existing results to highlight the research findings of RABV pathogenesis and strategies to manipulate the host cytoskeleton using various models of *in vitro* and *in vivo* studies. These results have also been categorized in different subheadings to segregate similar findings under one subheading. Materials in a language other than English were excluded, while unindexed journals, websites, thesis reports, and magazine reports were also excluded (20).

## Results

### Evasive Strategies of RABV With Cytoskeleton

Keeping in view the uniform integrity of the cytoskeleton in the neurons, it is believed that neurodegenerative diseases like rabies influence the dynamics, structure, and gene expression of the cytoskeleton and related proteins. Several studies support the fact that breach and discontinuity in the cytoskeleton network (microtubules and filamentous actin) develop neurodegenerative diseases, and rabies is one of the leading infectious diseases of the nervous system (14). RABV has many ways to manipulate the cell cytoskeleton to induce pathological mechanisms that have been poorly understood so far. However, the followings are the most prevailing, but diversified sets of information that countercheck the host defense pathways using a variety of RABV-cytoskeleton interactions. These approaches also demonstrate that RABV sneakily exploits the actin and microtubule cytoskeleton for efficient viral replication, transcription, and intracellular transport.

### RABV Alters the Gene Expression of Cytoskeleton-Related Proteins

Proteomic studies and real-time PCR assays quantify the viral proteins that aid molecular biologists and virologist to investigate the potential therapeutic agents, biomarkers, and foresee the generalized pathogenic pathways of the viruses (19). It has now been well established through *in vivo* and *in vitro* studies that RABV and related genotypes modulate the gene expression of cytoskeleton-related proteins in a variety of cell lines. Even humans nerve cells showed disturbances in the biological pathways of cytoskeleton organization or dynamics which control retrograde axonal transport, synaptic activity, and signaling events (4). The ingenuity pathways analysis shows that the RABV infected neurons in humans display abrupt alterations in the genetic expression of several key cytoskeleton-related proteins that regulate the intactness and structure of actin and microtubule cytoskeleton. Some of the notable genes increase the normal functioning of the cells which include calpastatin (CAST) and cyclin-dependent kinase inhibitor 1B (CDKN1B), while regulatory subunit 1 of the cyclin-dependent kinase (CDK5R1), clip associating protein 1 (CLASP1), and cAMP-responsive element-binding protein 1 (CREB1dec) decrease the cellular functions. Only doublecortin-like-kinase 1 (DCLK1) causes variable changes in the cellular functions. All of these genes are involved in the regulation and release of neurotransmitters, cell growth and differentiation, and stabilize microtubules (21). Another proteomic analysis shows the higher expression levels of tubulin, tropomyosin, and vimentin which are involved in the synthesis of elongated microtubules, development of axonal growth cones of filopodia (cytoplasmic projections larger than lamellipodia) or lamellipodia (thin membranous projections or protrusions of motile cells), and reinforce cytoskeleton to avoid external stress (22). The vimentin belongs to the intermediate filaments of the cytoskeleton, and preserves the cellular morphology by maintaining cytoskeleton shape, while tropomyosin also adjusts muscular contractions with the help of actin filaments (4, 14). Few studies of differential gene expression describe the abnormal neuronal structures due to the aberrations in the cytoplasmic proteins, and their corresponding pathways under RABV infections (22–24). Of these proteins, actin-related protein 2/3 complex subunit 3 and actin-related protein 2 showed higher gene expression, and the first one is meant for the actin polymerization, while the latter one maintains cellular polarity through the organization of the cytoskeleton in synaptosomes (24). However, neurofilament light polypeptide shows lower gene expression because it controls the axonal transport and aligns the assembly of axonal bundles (24). These are the salient proteins, and yet there are other actin-associated proteins (alpha-actinin-1 that anchors actin to various cellular structures, dynein light chain (DLC), capping proteins, and drebrin that generate stabilized actin filaments concentrated in the dendritic spines) and microtubule-associated proteins (microtubule-associated proteins-6) which are abundantly concentrated in the synaptosomes (5, 15, 24). The CVS strain of RABV also changes the 7 host proteins including the vimentin, stathmin, and capping proteins of the cytoskeletal in baby hamster kidney cell line (25). Interestingly, the protein-protein interactions discover that RABV transforms multiple neurochemical pathways related to synaptic plasticity, and nerve impulse transmission by renovating the organized proteins of the cytoskeleton (24, 26). A study also highlights the upregulation of the gene expression pattern of important microtubule-related proteins in cortical neurons of mouse brain tissue (27).

Microtubule-associated protein-2 (MAP-2) is a fundamental protein required to sustain neuronal shape and structure through the polymerization of dynamic microtubules. Most importantly, it also cross-links many biological pathways of neurotransmitters and maintains the assembly of other molecular trails involving different proteins of the cytoskeleton components (5, 28, 29). The downregulation pattern in the MAP-2 has already been observed under pathogenic infection of RABV (28, 30). In another study, RABV increases the genetic expression of NF-H (a protein that controls axonal growth) and MAP-2 which are integral proteins for the growing ends of tubulins as well (31), even though previously a proteomic approach reveal the similar findings in RABV infected human pyramidal nerve cells (32). A CVS strain of RABV decreases the gene expression of more than 90% of the cytoskeleton-related genes while increasing the genetic expression of 39 important genes in mouse brain tissue (33). Keeping in view the importance of microtubule and actin-related proteins, end binding protein 3 (EB3) is one of the basic proteins that maintain microtubule-based signaling pathways, and also shapes the polar ends of the tubules, whereas p140cap protein controls actin dynamics and interacts with EB3 to optimize morphology of dendritic spines (29). Dendritic spines are the tiny protrusions at the end of dendrites that are abundant in other actin-binding proteins, namely, cortactin, drebrin, and CaMKIIβ. The cortactin performs actin polymerization and the rearrangement of actin filaments under the cell cortex, while CaMKIIβ controls neuronal growth and nerve plasticity with the help of the binding module of actin filaments (15, 34). Two different strains (the street and fixed RABVs) also downregulate the gene expression of EB3 and p140cap in cultured neuronal cells (29). These changes demonstrate that imbalance in the biochemical and neuronal homeostasis may lead to developing these degenerative changes in neuronal cells (14). The failure in the repair of the cytoskeleton network may lead to uncertainty in the maintenance of intact neuronal physiology which perhaps creates cellular dysfunctions (31, 35).

### RABV Depolymerizes Microtubule and Actin

Rabies virus (RABV) causes depolymerization of microtubules by reducing the acetylated a-tubulin and this process facilitates the formation of RNA particles, while the M protein of RABV also up-regulates the gene expression of histone deacetylase 6 (HDAC6) which is a major structural protein to optimize actin and microtubule dynamics through the deacetylation of α-tubulin and cortactin. Moreover, the inhibition of HDAC6 also reduces the formation of RNA synthesis (36). The degenerative changes in the axons and dendritic tree are also caused by different strains of RABV. A recent study has shown that axonal degeneration occurs due to the depletion of nicotinamide adenine dinucleotide which is an energy producer molecule and accomplishes the proteolytic breakdown of MAP-2 and neurofilaments by calpain in extrinsic compartmental-nerve culture system (37).

*In vivo* and *in vitro* experiments show that street strain (MRV) of RABV causes dendritic injuries and depolymerization of actin filaments as dendritic spines are mostly enriched with filamentous actin (38). The decrease in the ratio between globular and filamentous actin in RABV-infected cells had also been calculated using western blot analysis (38). The alteration in neuronal actin was correlated with neuronal dysfunction. RABV depolymerizes the neuronal cytoskeleton by decreasing the actin bundle formation (39). Concisely, the N protein of RABV inhibited bundle formation of neuronal actin by inducing the dephospho-synapsin I which was an actin regulatory protein found in neurons. This was also experimentally demonstrated in confocal microscopy which showed the decreased intensity of immunofluorescence staining in the filamentous actin of neuroblastoma cells under RABV infection (40). A similar study was also conducted in different cell lines to observe the kinetics of focal adhesion kinase (FAK) and P protein of different RABV genomes. The FAK controlled the dynamic actin cytoskeleton through several actin-binding proteins. It also delimited the turnover rate of actin monomers in actin polymerization. Consequently, the decreased staining of filamentous actin was observed (41).

### Clathrin-Mediated Endocytosis of RABV Requires Intact Actin

The G protein of RABV follows clathrin-mediated endocytosis for the receptor-mediated attachment and entry in the endothelial and vero cells with the help of actin filaments and associated proteins (35, 42–45). In vero cells, the internalized particles of RABV rely on microtubules for their motility as observed by the colocalization of SRV9 (an attenuated strain of RABV) with microtubules in immunofluorescence. Likewise, another study depicts similar results in which RABV is internalized in the peripheral neuronal cells (42). Vesicular stomatitis virus expressing recombinant G protein of RABV is experimentally internalized in the vero cell line using partially coated pits of clathrin-mediated endocytosis with the help of actin nucleation. The kinetics of G protein expressing viral particles is almost similar to vesicular stomatitis virus with respect to the time of attachment and association of pathogenic particles with the kidney cells (3). Similarly, different treatments of drugs that block or disrupt the clathrin-coated vesicles also inhibit the entry of RABV inside the cells. These drugs include sucrose, methyl-beta-cyclodextrin, and chlorpromazine, while the chlorpromazine also blocked the Australian bat lyssavirus G protein-mediated entry in HEK293T cells (3, 43). Latrunculin inhibits actin polymerization in the clathrin-coated pits. This drug also lessened the entry of Australian bat lyssavirus in HEK293T cells up to 62% (3). Similar types of drug tests verify that RABV does not follow the caveolar-dependent endocytosis (35, 43).

**Figure 3:**
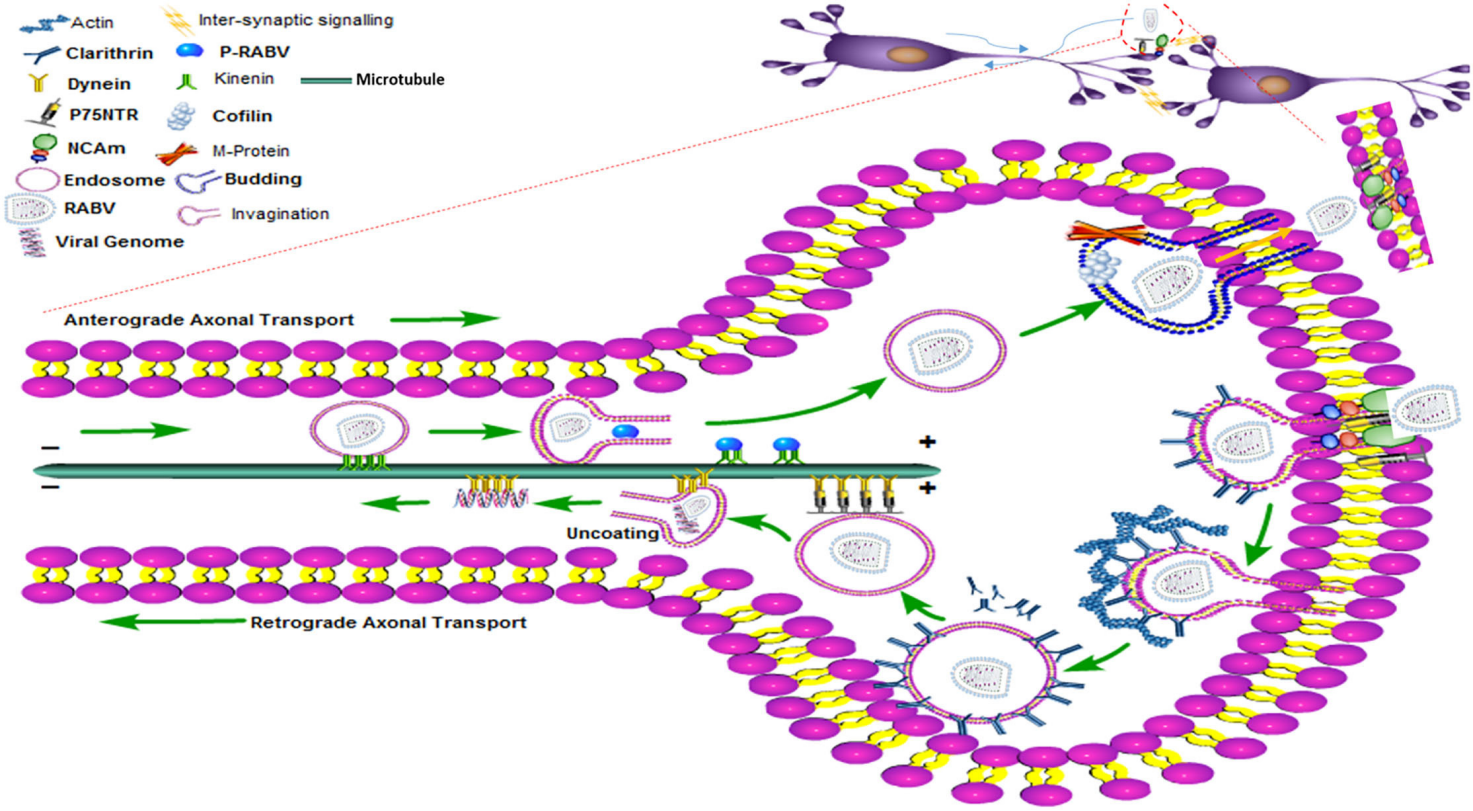
The clathrin-mediated endocytosis and the transport (anterograde and retrograde) pathway of RABV within the nerve cell showing motor proteins, entry and exist paths of the RABV with the help of the cytoskeleton network.

Nevertheless, experiments with HEP-2 and BHK-21 cells demonstrate that RABV might also employ additional passageways to enter the non-neuronal cell types (45, 46). For viruses, actin polymerization is an active process during receptor attachment and endocytosis. Therefore, further studies regarding drug experiments are needed to explore different cell types, interactions of RABV receptors, and the structure of the cytoskeleton elements so that a trajectory pathway can be envisaged (47). Cofilin is a well-known binding protein of actin and controls the remodeling and dynamic states of actin filaments. Remarkably, the M protein of RABV upregulates phosphorylated cofilin and promotes viral budding which is again dependent on the actin cytoskeleton (47). Hence, the internalization and budding of RABV require the assistance of actin and actin-based cytoskeleton (48). The trans-synaptic transmission of RABV has been shown in the diagram (Figure 3) where RABV is attached to the synaptic membrane *via* receptors, P75NTR and NCAM with the help of the G protein of RABV. The process of internalization is mediated by the clathrin protein, clathrin-coated pits, and actin proteins while the actin has been densely populated beneath the cell membrane or in the synaptic terminal. The legend termed “invagination” shows that the RABV is coated within the clathrin-coated vesicle and formed with the surrounding actin filaments. As the clathrin protein uncoats, either the full genome is encapsulated within the endosome or the chelates of P/N protein are taken up by the dynein motor protein. The P and N proteins of RABV now interplay with the DLC to propagate the viral trafficking and transport it toward the cell body using the microtubule as tracks. All the process of retrograde transport has been diagrammed beneath the single microtubule. The RABV completes the transcription and replication within the nucleus (not shown), it then moves back to initiate its exit pathway once again using the stable tracks of microtubules. This reverse transport of the genome is designated as anterograde transport of the RABV which is accomplished by the kinesin motor proteins. These kinesin proteins harbor the fully replicated genome or the naked G protein of RABV and slide toward the cell periphery or the positive directed end of the microtubule to start the process of budding. The budding phenomena need the phosphorylated cofilin and M protein of RABV to exit and join the next cycle of infection.

### RABV Requires Microtubules for Intracellular Trafficking

Viruses recruit motor proteins with the help of accessory proteins and employ the tracks of microtubules as pathways for the anterograde and retrograde transport of whole or partial viral parts for successful completion of the life cycle (12, 13). The intracellular transport of viruses is a complex system where the cytoplasmic environment is heavily crowded with various signaling and accessory proteins (7). In this situation, the microtubules act as polarized roads for the transport of viral cargoes to specific cellular destinations, while kinesins and dynein motor proteins act as energy vehicles to do the job of transportation of essential molecules from the plus or minus end of the microtubules to the other ends (49). Since cellular organelles are larger in size, therefore, energy is required to overcome the barriers of diffusion right after the internalization. Experiments conducted with the fluorescent-labeled G protein of RABV have shown long-range retrograde transport of virion using microtubules in neuroblastoma cells (6, 50, 51).

The mixed polarity of these microtubules in dendritic spines ensures the slow and fast axonal transport (adopted for RABV) inside the cells depending upon the availability of motor proteins in the absence of antiviral drugs, while plus ends are usually laid out in the axonal shaft as compared to the dendritic microtubules (12, 13). This fact has also been well established that RABV uses tracks of the microtubules for long-distance bidirectional transport with the help of motor proteins (7). In experiments in which the microtubule depolymerizing drugs such as vinblastine, nocodazole, and colchicine were used, the flow in the transport of various viruses (including RABV) was disrupted (6, 49).

Dynein deficient drosophila neurons produce transport defects leading to the misguided minus-end directed microtubules in axons (12). This suggests that the dynein motor is the most significant protein for retrograde axonal transport (6). The DLC is a microtubule minus-end-directed motor protein for retrograde transport of various molecular cargoes and organelles and also interacts with the actin in the early phase of RABV infection (11, 49). Modifications in the dynamics and spatial organization of microtubules play a critical role in the prognosis of infection. The P protein of RABV is a complex hub of protein with multiple binding sites that carries vital and multi-molecular interactive features in host-pathogen mechanisms including the cytoskeleton components. The structural or truncated forms of the P protein are p2, p3, p4, and p5 of which p3 has been extensively explored. The p3 interacts with the DLC in 2 different ways (52, 53). The p3 interacts with the DLC8 through the DLC association sequence that triggers the movement of the dynein motor proteins along the length of the microtubule, the second way is the formation of stable attachments with the microtubules with the help of microtubule-associated sequences (7).

### Microtubules and Actin Are Required for RABV Inclusion Bodies

Nocodazole inhibited the dynamics and growth of microtubules. The RABV-infected BSR cells (cloned cells from the BHK-21 cell line) were treated with this drug that prevented the formation of “late” negri body-like inclusion bodies in the cells (40), while in another study, the same drug also diminished a few small size inclusion bodies. These negri bodies are viral replication machinery and eject the RNP complex which is independent of the microtubule or actin network, but the transport of RNP is carried out with the help of microtubules. Moreover, the nearby movement of the RNP complex to negri bodies was restricted due to the inhibitory effect of the drug that ruptured or destabilized the tubulin filaments (43, 54). In the same way, the contribution of actin was also investigated by using cytochalasin D drug that depolymerizes actin dynamics. As a result, the inclusion bodies appeared smaller and scrappy in the cytoplasm (54).

Due to the overwhelming cellular stress, and overburdened viral replications, the misfolded viral aggregates are sequestered in the concentrated areas of the cytosol that give rise to ubiquitinated proteins which are known as aggresomes. These aggresomes structurally resembled some extent the negri bodies and are located at the microtubule organization center, but negri bodies are not covered externally with the microtubule protein vimentin. The negri bodies are also not connected to the microtubule organization center. These aggresomes are transferred to the inclusion bodies *via* microtubules using dynein-dependent transport. Microtubule inhibitory drug treatment increased the size and number of negri bodies per cell which further evidenced the fact that polymerization of microtubules coordinates the composition of negri bodies (55). The treatment of nocodazole also impaired the trajectories pathways, movement pattern, and velocities of viral particles which prove that microtubules are necessary for the intracellular transport of RABV (43).

### The Interactions of N, P, and L Proteins of RABV With Cytoskeleton

Being a neurotropic virus, the intracellular transport of RABV depends on long-distance travel on axonal pathways. However, the approach by which RABV steals host cell transport machinery is equivocal. One strategy is the exploitation of host actin-microtubule cytoskeleton for invasion, and short or long-range trafficking at the cellular periphery even to reach the perinuclear region. The intracellular trafficking and transport of RABV rely on the stability of microtubule and actin filaments (56).

For example, microtubule and microtubule-associated proteins are involved in antiviral response by inducing interferon release that counters RABV intracellular replication. RABV handles this host cell strategy through its P protein by inhibiting the interferon production, and JAK/STAT signaling pathway which again depends upon the stability of the microtubules for intracellular trafficking of essential pathway building signaling proteins and molecular cues (8, 9, 52). Thus, in this way, the P protein is involved in the inhibition of IFN and promotes intracellular viral replication (9).

The P protein of RABV was also investigated using the FAK as an essential molecular ingredient in the yeast two-hybrid system. The dimerization domain of P protein was 106, while 131 was meant for the C-terminal domain of FAK. It has been well established that FAK interacts with the P protein of RABV to cause RABV infection in infected host cells. The P protein and FAK were experimentally colocalized and coimmunoprecipitated in negri bodies, and this experimental interaction between FAK and P protein was also reconfirmed through downregulation of FAK, which finally inhibited the expression of P protein of RABV. Hence, FAK is an imperative constituent for RABV pathogenesis and an important cellular partner of the P protein of RABV (41).

The P protein of all genotypes of RABV also interacts with the light chain of dynein motor protein (53, 57) and the RABV inclusion bodies are also closely in contact with the filamentous actin underneath the cell membrane or nearby the endocytic entry during the earlier events inside the cells. Thus, the light chain possesses a dual interactive role with the cytoskeleton components for the transport of the RNP complex in neuronal cells (53). DLC1 binding motif in L protein of RABV mediates microtubule binding with the help of dynein motors. As the motif was disrupted, the microtubule localization in the cell was disrupted. The L protein of RABV also regulates the post-translational modifications of the microtubule cytoskeleton. Hence, the microtubule is an essential structural element in controlling the intracellular transport (57).

The oligomeric state of P protein of RABV in the interactive process of more than one polypeptide chain of the protein gives rise to quaternary protein structure. The P protein has the unique ability to interact with the microtubules either through a microtubule-facilitated or inhibitory mechanism. Interestingly, the P protein can switch between these two states with the help of interferon-activated transcription factor STAT1 to regulate trafficking and motor-based bidirectional transport (9).

On the other hand, the N protein of RABV does not require the support of the cytoskeleton for its synthesis in the neuronal and non-neuronal cell cytoplasm of dorsal root ganglia (58), but the fluorescent-tagged L protein of RABV does interact with the microtubule cytoskeleton and facilitates the production of tubulin to enhance viral infection and transport. Further analysis also shows that the binding motif of DLC1 in the L protein of RABV can bind with the microtubules. As binding of P and L protein of RABV with DLC1 is valuable for transcription, both of these protein motifs regulate the gene expression of DLC1 inside the cells. The overexpression of DLC1 also enhances the tubulins' growth and maintenance to facilitate viral infections. The silencing or downregulation of the motif in the L destabilizes the microtubule and hence, the DLC1 is significantly associated with the L gene of RABV for smooth transport functions through the microtubule (59).

Two-dimensional far-western blotting confirmed that the M protein of RABV also interacts with six different proteins in rat brain stem including the actin cytoplasmic-1 that was verified in co-immunoprecipitation. All these proteins regulate vital cellular processes as described earlier and the findings were also verified in mouse neuro-N2a cells (60).

## Conclusions

The review demonstrates a comprehensive analysis of various research studies to show that RABV attempt to dysfunction the axonal transport, structure of the neurons, and neurotransmitters in the synaptic terminal by modulating the cytoskeleton network. Subcellular transport is the best level of investigation to explore the individual lines of evidence used by the viruses. RABV sneakily exploits actin, microtubules, kinesin, and dynein motor proteins for its survival. However, the functional consequences and pathological outcomes are yet to be elucidated through experiments conducted through fluorescent-tagged proteins or cargoes in live cell imaging. The prospects of new research directions, identification of the existing gaps, and inconsistency in the development of new research insights were also identified. The review has highlighted that the host cytoskeleton provides tracks and assistance in each step from the entry of the RABV until its budding. This review will help scientists and virologists to further elucidate the pathogenesis of RABV and define new research questions to generate possible hypotheses. Further research is needed to address how RABV subverts the cellular motor proteins to complete a successful cycle of infection. Scientific reconsiderations of all the experimental studies and molecular approaches can provide helpful clues in determining the pathogenesis of RABV inside the host cell. It will also reveal the functional morphology of cytoskeleton elements that possibly aid RABV in disrupting the normal cellular physiology. The futuristic approaches are required to develop which should be based on the available findings and analysis of the reviews. Few helpful strategies include the development of therapeutic agents, biotechnological procedures, preventive drugs, and attenuated viruses for the production of efficient vaccines. The role of actin and microtubule-binding proteins, especially cofilin needs to be further explored in the pathogenesis and budding of RABV to verify whether RABV directly or indirectly interacts with actin.

## Author Contributions

XL and CG conceived the idea, designed the study plan, and managed funding. ZN and SAl collected the literature review and extracted meaningful contents. TJ, SAh, and WA wrote the manuscript. MAN and MUS drew the images. MAI and AA revised the manuscript, added further contents, and improved the language. All authors read and approved the final version of the manuscript.

## Funding

This work was supported by the grants from the project Preclinical research and development of colon cancer-targeted drug rCCK8PE38KDEL (No. 20190304043YY).

## Conflict of Interest

The authors declare that the research was conducted in the absence of any commercial or financial relationships that could be construed as a potential conflict of interest.

## Publisher's Note

All claims expressed in this article are solely those of the authors and do not necessarily represent those of their affiliated organizations, or those of the publisher, the editors and the reviewers. Any product that may be evaluated in this article, or claim that may be made by its manufacturer, is not guaranteed or endorsed by the publisher.
